# Subcutaneous Levodopa Infusion for Parkinson's Disease: 1‐Year Data from the Open‐Label BeyoND Study

**DOI:** 10.1002/mds.28758

**Published:** 2021-09-08

**Authors:** Werner Poewe, Fabrizio Stocchi, David Arkadir, Georg Ebersbach, Aaron L. Ellenbogen, Nir Giladi, Stuart H. Isaacson, Karl Kieburtz, Peter LeWitt, C. Warren Olanow, Tanya Simuni, Astrid Thomas, Abraham Zlotogorski, Liat Adar, Ryan Case, Sheila Oren, Shir Fuchs Orenbach, Olivia Rosenfeld, Nissim Sasson, Tami Yardeni, Alberto J. Espay, and for the BeyoND study group

**Affiliations:** ^1^ Department of Neurology Medical University Innsbruck Innsbruck Austria; ^2^ University and Institute for Research and Medical Care IRCCS San Raffaele Rome Italy; ^3^ Department of Neurology, The Faculty of Medicine, Hadassah Medical Center Hebrew University of Jerusalem Jerusalem Israel; ^4^ Movement Disorder Clinic Beelitz‐Heilstaetten Beelitz Germany; ^5^ Michigan Institute for Neurological Disorders Farmington Hills Michigan USA; ^6^ Tel Aviv Medical Center, Sackler School of Medicine, Sagol School of Neurosciences Neurological Institute, Tel‐Aviv University Tel Aviv Israel; ^7^ Parkinson's Disease and Movement Disorders Center of Boca Raton Boca Raton Florida USA; ^8^ Departments of Neurology Wayne State University School of Medicine and Henry Ford Hospital Detroit Michigan USA; ^9^ Clintrex Research Corp Sarasota Florida USA; ^10^ Northwestern University Feinberg School of Medicine Chicago Illinois USA; ^11^ Department of Neuroscience, Imaging and Clinical Sciences and Center of Advanced Studies and Technology CAST University Chieti Pescara Chieti Italy; ^12^ Department of Dermatology, The Faculty of Medicine, Hadassah Medical Center Hebrew University of Jerusalem Jerusalem Israel; ^13^ NeuroDerm Ltd Rehovot Israel; ^14^ James J. and Joan A. Gardner Center for Parkinson's Disease and Movement Disorders University of Cincinnati Cincinnati Ohio USA

**Keywords:** infusion, levodopa, ND0612, Parkinson's disease, safety, subcutaneous

## Abstract

**Background:**

Continuous, subcutaneous (SC) levodopa/carbidopa infusion with ND0612 is under development as a treatment for patients with Parkinson's disease (PD) and motor fluctuations.

**Objective:**

Evaluate 1‐year safety data.

**Methods:**

BeyoND is an open‐label study evaluating the long‐term safety of two ND0612 dosing regimens.

**Results:**

Of the 214 enrolled patients (24‐hour SC infusion: n = 90; 16‐hour SC infusion: n = 124), 120 (56%) completed 12 months of treatment. Leading causes for study discontinuation were consent withdrawal (19.6%) and adverse events (17.3%). Rates of discontinuation were reduced from 49% to 29% after a protocol revision and retraining. Systemic safety was typical for PD patients treated with levodopa/carbidopa. Most patients experienced infusion site reactions, particularly nodules (30.8%) and hematoma (25.2%), which were judged mostly mild to moderate and led to discontinuation in only 10.3% of the participants.

**Conclusions:**

Subcutaneous levodopa/carbidopa continuous infusion with ND0612 is generally safe, with typical infusion site reactions for SC delivery as the main adverse event. © 2021 The Authors. *Movement Disorders* published by Wiley Periodicals LLC on behalf of International Parkinson and Movement Disorder Society

ND0612 (NeuroDerm Ltd, Rehovot, Israel) is an investigational subcutaneous (SC) delivery system providing minimally invasive, continuous infusion of liquid levodopa/carbidopa for treating patients with Parkinson's disease (PD) who experience motor fluctuations. Previous studies have shown that ND0612 provides stable plasma levodopa concentrations,^1^ significantly reduces daily OFF time,^1^, ^2^ and increases ON time without significant dyskinesia.^2^ The primary aim of this study was to assess the long‐term safety and tolerability of ND0612 over 12 months of treatment, with a particular focus on infusion site reactions (ISRs) that are often associated with SC drug administration.

## Methods

### Study Design and Patient Population

The BeyoND study is an ongoing, international, multicenter, 102‐month, open‐label study evaluating the long‐term safety and tolerability of two ND0612 dosing regimens in PD patients experiencing motor fluctuations. The trial is conducted in accordance with the Declaration of Helsinki; study protocols and amendments were approved by the ethics committee at each site and all patients provided written informed consent. The study is registered at ClinicalTrials.gov (NCT02726386).

The study population includes 21 patients who had completed a prior randomized, open‐label, 28‐day study^2^ and consented to roll‐over into the long‐term safety study. Additional patients could be added who were ≥30 years, had a Hoehn‐Yahr score ≤3 during ON, experienced OFF time ≥2 hours per day, and had predictable early morning OFF periods. Patients had to be receiving stable doses of standard oral levodopa (≥4 oral doses per day or ≥3 doses/day of extended‐release levodopa/carbidopa [Rytary, Impax Pharmaceuticals, USA]) and at least one additional PD treatment. Full inclusion and exclusion criteria are provided in Table S1.

The study was initiated in May 2016 and patients were assigned to the following regimens:

1. 24 hours infusion: fixed day rate of up to 0.64 mL/h for 18 hours, followed by a night rate of 0.08 mL/h for 6 hours to deliver a total daily dose of up to 720/90 mg of levodopa/carbidopa. All patients who had been previously assigned to the 24‐hour group in the prior study^2^ continued on this dosing regimen; patients who had previously been assigned to the 14‐hour daytime regimen were switched to the 24‐hour regimen.
2. 16‐hour “waking hours” regimen: fixed rate of 0.75 mL/h to deliver a total infusion dose of 720/90 mg of levodopa/carbidopa over 16 hours. The device is removed at night and patients in this group also receive a morning oral dose of levodopa/carbidopa upon awakening.

Patients and their study partners were trained and assisted in their homes on the proper operation of the pump system during the first week of treatment by a home nursing service. ND0612 was administered using two SC infusion sites, with daily rotation of sites; patients in the waking day regimens removed the pump nightly after completing treatment for 16 hours or at bedtime (whichever came first).

In March 2018, in response to a higher than expected study dropout rate, a protocol amendment increased enrollment to 210 patients, provided education on expected treatment‐emergent adverse events (TEAEs), and increased home nursing support. Simultaneously, additional investigator training was implemented to emphasize patient selection, education, and strategies to reduce ISRs.

### Assessments

Clinic visits were scheduled at week 1, and months 1, 2, 3, 4, 6, 9, and 12. Safety outcomes were performed at every visit and included recording of TEAEs, with specific attention to ISRs including a visual analog scale (VAS, 0–100 mm) for infusion site pain assessment. Other safety measures included standard laboratory parameters, vital signs, physical and neurological examination, the Columbia Suicide Severity Rating Scale (C‐SSRS),^3^ the Epworth Sleepiness Scale (ESS),^4^ and the Questionnaire for Impulsive‐Compulsive Disorders (ICDs) in PD‐Rating Scale (QUIP‐RS).^5^Home diaries of motor function (at months 1, 3, 6, 9, and 12) were used to record “good” ON time (sum of ON time without dyskinesia and ON time with non‐troublesome dyskinesia), OFF time normalized to 16 waking hours.^6^, ^7^ Additionally, Unified Parkinson's Disease Rating Scale (UPDRS) Part 2 (activities of daily living, ADL), UPDRS Part 3 (motor), and Clinical Global Impression of Change (CGI‐C) were performed.^8^

### Statistical Analyses

No formal sample size calculation for this safety study was performed. However, a sample size of 210 treated patients was considered sufficient^9^ for evaluating the 1‐year safety and tolerability of ND0612. All efficacy measures were considered exploratory. A multivariate logistic regression (stepwise selection algorithm) was performed to detect the most important baseline factors explaining early discontinuation; variables with a *P* value <0.2 were retained in the model.

## Results

### Patient Flow and Baseline Characteristics

Of the 276 patients screened, 214 patients were enrolled (24‐hour dosing regimen: n = 90; 16‐hour dosing regimen: n = 124) at 46 sites in eight countries (Figure S1). One hundred and twenty patients (56.1%) completed 12 months in the study and 94 (43.9%) terminated early, with a similar proportion of early terminations between the two regimens. Leading causes for premature discontinuation over the course of the study were consent withdrawal (n = 42, 19.6%) and AEs (n = 37, 17.3% most of which discontinued before day 150). Concomitant catechol‐O‐methyltransferase (COMT) inhibitor use and low baseline body mass index (BMI) (<20) were each associated with higher rates of discontinuations (odds ratio [OR] OR = 2.3, *P* = 0.01 and OR 3.15, *P* = 0.03, respectively) Rates of discontinuation decreased from 49% to 29% for patients enrolled after the March 2018 protocol amendment and increased site training on ISRs. Patient characteristics for both dosing regimens are shown in (Table 1).

**TABLE 1:** Patient characteristics

Variable	24 h/d regimen (N = 90)	16 h/d regimen (N = 124)	Total (N = 214)
Age (years); mean ± SD	64.2 ± 8.9	63.9 ± 8.9	64.0 ± 8.9
<65 y	43 (47.8)	63 (50.8)	106 (49.5)
≥65 y	47 (52.2)	61 (49.2)	108 (50.5)
Sex (female/male); n (%)	32 (35.6)/58 (64.4)	40 (32.3)/84 (67.7)	72 (33.6)/142 (66.4)
Ethnicity; n (%)			
Caucasian	86 (95.6)	116 (93.5)	202 (94.4)
Other	4 (4.4)	8 (6.5)	12 (5.6)
BMI (kg/m^2^); mean ± SD	27.0 ± 5.5	27.2 ± 5.9	27.1 ± 5.7
<20	11 (12.2)	11 (8.9)	22 (10.3)
≥20	79 (87.8)	113 (91.1)	192 (89.7)
Modified Hoehn & Yahr; n (%)			
<2	4 (4.4)	5 (4.0)	9 (4.2)
2	37 (41.1)	52 (41.9)	89 (41.6)
2.5	17 (18.9)	32 (25.8)	49 (22.9)
3	32 (35.6)	35 (28.2)	67 (31.3)
MMSE total score; mean ± SD	28.8 ± 1.2	28.8 ± 1.2	28.8 ± 1.2
Time since PD diagnosis (y); mean ± SD	10.6 ± 5.3	7.9 ± 3.8	9.0 ± 4.7
Time since onset of fluctuations (y); mean ± SD	5.3 ± 4.3	5.2 ± 4.2	5.3 ± 4.2
Total daily levodopa; mean ± SD			
Dose (mg)	1090 ± 623	1004 ± 540	1040 ± 577
Frequency	5.9 ± 2.2	5.1 ± 1.7	5.5 ± 2.0
Concomitant medications; n (%)			
Dopamine agonists	52 (57.8)	58 (46.8)	110 (51.4)
MAO‐B inhibitors	37 (41.1)	44 (35.5)	81 (37.9)
COMT inhibitors	28 (22.6)	24 (26.7)	52 (24.3)
Amantadine	25 (27.8)	30 (24.2)	55 (25.7)

Abbreviations: h, hour; d, day; y, year; SD, standard deviation; BMI, body mass index; MMSE, Mini‐Mental State Examination; PD, Parkinson's disease; MAO‐B, monoamine oxidase B; COMT, catechol‐O‐methyltransferase.

### Safety Analyses

Over 1 year, the median individual ND0612 exposure was 337 days (range 2–387 days), without major differences between the 24‐hour and 16‐hour dosing regimens. Most patients (85.5% across dosing regimens) reported ≥1 TEAE, mainly mild–moderate in severity, and without differences in incidence rates between the two dosing regimens (Table 2).

**TABLE 2:** Treatment emergent adverse events

Adverse event	24 h/d regimen (N = 90) n (%)	16 h/d regimen (N = 124) n (%)	Total (N = 214)
Any TEAE	78 (86.7)	105 (84.7)	183 (85.5)
Drug‐related TEAEs	65 (72.2)	78 (62.9)	143 (66.8)
Infusion site TEAEs	55 (61.1)	66 (53.2)	121 (56.5)
Serious TEAEs	17 (18.9)	14 (11.3)	31 (14.5)
Death	0	1 (0.8)	1 (0.5)
TEAEs reported in ≥5% of patients in any group			
Infusion site nodule	31 (34.4)	35 (28.2)	66 (30.8)
Infusion site hematoma	24 (26.7)	30 (24.2)	54 (25.2)
Infusion site infection	15 (16.7)	11 (8.9)	26 (12.1)
Fall	12 (13.3)	8 (6.5)	20 (9.3)
Infusion site pain	13 (14.4)	15 (12.1)	28 (13.1)
Dyskinesia	8 (8.9)	8 (6.5)	16 (7.5)
Infusion site erythema	9 (10.0)	7 (5.6)	16 (7.5)
Urinary tract infection	7 (7.8)	13 (10.5)	20 (9.3)
Back pain	5 (5.6)	3 (2.4)	8 (3.7)
Contusion	5 (5.6)	3 (2.4)	8 (3.7)
Infusion site edema	5 (5.6)	5 (4.0)	10 (4.7)
Nausea	3 (3.3)	15 (12.1)	18 (8.4)
Infusion site eschar	2 (2.2)	15 (12.1)	17 (7.9)
Anxiety	2 (2.2)	8 (6.5)	10 (4.7)
Depression	1 (1.1)	8 (6.5)	9 (4.2)
Fatigue	0 (0)	7 (5.6)	7 (3.3)
TEAEs leading to discontinuation			
Any TEAE leading to discontinuation	17 (18.9)	20 (16.1)	37 (17.3)
Infusion site reaction	11 (12.2)	11 (8.9)	22 (10.3)
Dyskinesia	2 (2.2)	1 (0.8)	3 (1.4)
Death^a^	0 (0)	1 (0.8)	1 (0.5)
Other^b^	4 (4.4)	8 (6.5)	12 (5.6)

^a^ One patient in the 16‐hour regimen died due to an unrelated cardiac event.

^b^ TEAEs affecting single patients only.

Abbreviation: TEAE, treatment emergent adverse effect.

In the first year, 37 patients (17.3%) terminated treatment prematurely due to TEAEs (most commonly because of ISRs (n = 22, 10.3%) and 3 (1.4%) due to dyskinesia. Approximately half of the patients terminating early due to TEAEs did so within the first 2 months of treatment (n = 8 in the 24‐hour regimen and n = 10 in the 16‐hour regimen).

The most frequent ISRs reported as a TEAE were infusion site nodules (n = 66, 30.8%) and infusion site hematoma (n = 54, 25.2%) (Figure S2), which were generally mild and resolved without sequalae. Twenty‐five patients (11.7%) had an infusion site infection, reported more commonly in the 24‐hour than the 16‐hour dosing regimen (16.7% vs. 8.1%, respectively). Mean pain VAS scores were similar for both regimens and did not exceed 20/100 mm at any time during the study. Sixteen patients (7.5%) reported increased dyskinesia as a TEAE, 8 patients within the first week.

Thirty‐one patients (14.5%) had at least one serious TEAE, with a similar incidence rate across both regimens. Serious TEAEs reported for more than one patient were infusion site infection (n = 5), ON–OFF phenomenon (n = 3), and rib fracture secondary to fall (n = 2 in the 24‐hour regimen). One patient had an impulsive compulsive disorder (ICD) (considered related to concomitant dopaminergic agonist use) and one patient in the 16‐hour regimen died due to an unrelated cardiac event. There were no observable trends for change in daytime sleepiness, suicidality, ICDs, laboratory safety measures, or electrocardiogram changes.

### Exploratory Efficacy Analyses

Adjusted means (LS‐means) of daily “good” ON time increased from baseline by 2.3 hours at month 3 in the 24‐hour regimen (n = 55) and 2.6 hours in the 16‐hour regimen (n = 101), accompanied by corresponding reductions in daily OFF time and maintained for at least 12 months (Figure S3). UPDRS‐motor and ADL scores improved within the first month with both ND0612 regimens and maintained thereafter. Investigators assessed the majority of patients as improved throughout the duration of the study.

## Discussion

Subcutaneous infusion of levodopa/carbidopa with ND0612 was generally safe based on 135 patient‐years of exposure accumulated during the first year of this long‐term safety study. Although ISRs were common, they were generally mild and typical for SC drug delivery^10^, ^11^, ^12^, ^13^, ^14^ – leading to discontinuation in 10% of the participants. Exploratory efficacy data indicated clinically relevant^15^, ^16^ adjusted mean increases in good ON time and reductions in OFF time. Overall, these results suggest a favorable risk/benefit trade‐off for most patients.

In general, we observed no major differences in safety or exploratory efficacy between the “24‐hour” and “waking day” regimens and there was no evidence for the development of pharmacological tolerance to either. As previously reported for SC apomorphine infusions^10^, ^11^ we confirmed that continued training on managing and minimizing ISRs is essential. Indeed, following improvements to the protocol and retraining of sites, patient retention improved from 51% to 71%.

While the majority of patients developed skin nodules, these were only reported as an AE in a third of cases when designated as clinically significant by the investigator. The higher rates of infusion site infection with the 24‐hour regimen predated protocol amendments requiring ISR training. The association between low baseline BMI (<20) (*P* = 0.03) with high rates of discontinuation in this study illustrates an important patient characteristic pertinent to selection of optimal candidates for continuous SC infusion therapy.

We recognize several important limitations of this study. This was an open‐label safety study limiting conclusions on efficacy. There was a higher than expected dropout rate, which improved following increased trial site training regarding appropriate patient selection, setting of patient expectations, and the practical management of ISRs. Consensus guidelines such as those that have been developed for other infusion therapies^17^ may be helpful if the ongoing Phase 3 data support ND0612 approval.

Additional safety data (beyond 12 months) is being collected, with some patients already in their fifth year of treatment. A double‐blind, double‐dummy pivotal efficacy trial (NCT04006210) evaluating efficacy, safety, and tolerability of ND0612 in a similar PD population is also currently ongoing.

## Author Roles

W.P., C.W.O., K.K., and S.O. conceived the study. W.P., F.S., D.A., G.E., S.I., N.G., P.L., T.S., A.T., and A.E. were involved in data acquisition. L.A., S.F.O., S.O., O.R., and T.Y. were involved in the conduct of the study. N.S. was responsible for data analysis. All authors were involved in data interpretation. W.P., F.S., R.C., and A.J.E. wrote the first draft of the manuscript and all authors critically reviewed the manuscript and approved the final draft. AZ was involved in data interpretation, critically reviewed the manuscript and approved the final draft.

## Financial Disclosures

W.P. reports receiving personal fees from AbbVie, AFFiRiS, AstraZeneca, BIAL, Boston Scientific, Britannia, Intec, Ipsen, Lundbeck, NeuroDerm, Neurocrine, Denali Pharmaceuticals, Novartis, Orion Pharma, Prexton, Teva, UCB, and Zambon. He receives royalties from Thieme, Wiley Blackwell, Oxford University Press, and Cambridge University Press and grant support from The Michael J. Fox Foundation, EU FP7, and Horizon 2020. F.S. reports honoraria and consulting fees from Britannia Pharmaceuticals, GlaxoSmithKline, Boehringer Ingelheim, Lundbeck, Orion, Novartis, Teva, Pfizer, and Zambon. A.Z. reports fees for consultancy from NeuroDerm Ltd. D.A. has no financial interests to disclose. G.E. reports advisory roles for AbbVie, BIAL, Biogen GmbH, Desitin Pharma, STADA Pharma, and Neuroderm; lecture fees for AbbVie, BIAL, Britannia, Desitin Pharma, Licher GmbH, UCB Pharma, and Zambon Pharma and royalties from Kohlhammer Verlag and Thieme Verlag. A.L.E. reports honoraria and consulting fees from Allergan, Ipsen, Revance, Lundbeck, Affiris, Acorda, Kyowa, Biohaven, Teva, and Adamas. N.G. serves as consultant to Sionara, NeuroDerm, Pharma2B, Denali, Neuron23 and Abbvie, Sanofi‐Genzyme, and Biogen. He receives royalties from Lysosomal Therapeutics (LTI) and payment for lectures at Abbvie, Sanofi‐Genzyme, and the Movement Disorder Society. He received research support from The Michael J. Fox Foundation, the National Parkinson Foundation, the European Union, and the Israel Science Foundation as well as from Teva NNE program, Biogen, and Ionis. He receives support from the Sieratzki Family Foundation and the Aufzien Academic Center in Tel‐Aviv University. S.H.I. reports honoraria for CME, consultant, research grants, and/or promotional speaker on behalf of Abbvie, Acadia, Acorda, Adamas, Addex, Affiris, Alexva, Allergan, Amarantus, Amneal, Aptinyx, Axial, Axovant, Benevolent, Biogen, Britannia, Cadent, Cala, Cerecor, Cerevel, Cipla, Eli Lilly, Enterin, GE Healthcare, Global Kinetics, Impax, Impel, Intec Pharma, Ipsen, Jazz, Kyowa, Lundbeck, Merz, The Michael J. Fox Foundation, Mitsubishi Tanabe, Neuralys, Neurocrine, Neuroderm, Parkinson Study Group, Pharma2B, Prilenia, Promentis, Revance, Roche, Sanofi, Sunovion, Sun Pharma, Supernus, Teva, Theravance, UCB, and Zambon. P.L. reports advisory roles for Abide, Acorda Therapeutics, Adamas, Biogen, Cavion, Denali, Intec Pharma, Jazz Pharmaceuticals, Lundbeck, Neurocrine, Mitsubishi NeuroDerm, Prexton, Revance, Sage, and US WorldMeds; lecture fees from US WorldMeds, Acorda, American Academy of Neurology, and Kyowa Hakko Kirin; and research grant support from Abide, Acorda, Amneal, Lundbeck, The Michael J. Fox Foundation for Parkinson's Research, Mitsubishi, NeuroDerm, Parkinson Study Group, Pharma 2B, Revance, Hoffmann‐La Roche, Sunovion, Sun Pharma, and US WorldMeds. He is the editor‐in‐chief of *Clinical Neuropharmacology*. C.W.O. and K.K. report owning shares in Clintrex which provides services for multiple pharmaceutical and biotech companies. T.S. has served as a consultant for Acadia, Abbvie, Accorda, Adamas, Allergan, Amneal, Aptinyx, Denali, General Electric (GE), Kyowa, Neuroderm, Neurocrine, Sanofi, Sinopia, Sunovion, Roche, Takeda, Voyager, US World Meds, Parkinson's Foundation, and The Michael J. Fox Foundation for Parkinson's Research; has served as a speaker and received an honorarium from Acadia and Adamas; is on the scientific advisory board for Neuroderm and Sanofi; and has received research funding from the NINDS, Parkinson's Foundation, The Michael J. Fox Foundation, Biogen, Roche, Neuroderm, Sanofi, Sun Pharma, Abbvie, IMPAX, and Prevail. A.T. reports consulting fees from Newron, Zambon, and NeuroDerm. L.A., R.C., and T.Y. are employed by NeuroDerm. N.S. provides biostatistical services to NeuroDerm. S.O., S.F.O., and O.R. were employed by NeuroDerm at the time of the study. A.J.E. has received grant support from the NIH and The Michael J. Fox Foundation; personal compensation as a consultant/scientific advisory board member for Abbvie, Neurocrine, Amneal, Adamas, Acadia, Acorda, InTrance, Sunovion, Lundbeck, and US WorldMeds; publishing royalties from Lippincott Williams & Wilkins, Cambridge University Press, and Springer; and honoraria from US WorldMeds, Acadia, Sunovion, the American Academy of Neurology, and the Movement Disorders Society.

## Appendix

**BeyoND study group**: Austria: Werner Poewe; Czech Republic: Ladislav Pazdera, Yuliya Rizova; France: Jean‐Philippe Azulay, Teodor Danaila, Luc Defebvre, Franck Durif, Isabelle Roullet‐Solignac; Germany: Georg Ebersbach, Jan‐Rainer Kassubek, Siegfried Muhlack, Johannes Schwarz; Israel: David Arkadir, Ruth Djaldetti, Tanya Gurevich, Sharon Hassin, Ron Milo, Gilad Yahalom; Italy: Angelo Antonini, Ubaldo Bonuccelli, Elisabetta Gasparoli, Marco Onofrj, Fabrizio Stocchi; Poland: Malgorzata Krawczyk, Konrad Rejdak, Monika Rudzińska; USA: Jason Aldred, Cletus Aralu, Perminder Bhatia, Victor Biton, Barry Cutler, Aaron Ellenbogen, Alberto Espay, Stephen Flitman, Ramon Gil, Paul Ginsberg, Jeffrey Greenberg, Robert A. Hauser, Arnaldo Isa, Stuart Isaacson, Olga Klepitskaya, Kevin Klos, Rajeev Kumar, Maureen Leehey, Peter LeWitt, Neepa Patel, Beth Safirstein, Joseph Savitt, Tanya Simuni, Lynn Struck, Danette Taylor, Karen Thomas, Daniel Truong, Alberto Vasquez.

## Supporting information


**Appendix S1**. Supplementary Information

## Data Availability

NeuroDerm will share the data from this study with qualified researchers who provide a valid research question and sign a data access agreement. Address proposals to info@neuroderm.com.
