# Supplementary material for: Subcutaneous Levodopa Infusion for Parkinson's Disease: 1‐Year Data from the Open‐Label BeyoND Study
Source: Mov Disord. 2021 Sep 8;36(11):2687–92. doi: 10.1002/mds.28758 (PMC9291977; doi:10.1002/mds.28758)
Supplement: Supplementary file 1 — Appendix S1. Supplementary Information [file MDS-36-2687-s001.pdf]

Appendix

Supplementary Table e1. Inclusion and exclusion criteria

Inclusion criteria	Exclusion criteria
For patients from 006 study (Olanow et al, 2021)<br>• Completed the treatment period of study ND0612-006 not more than one month prior to enrolment.<br>• Willing and able to administer the SC infusion alone or with the assistance of a study partner and able to comply with the study specific procedures.<br>• Able to and has signed an Institutional Review Board/Ethics Committee - approved informed consent form.	For patients from 006 study<br>• Previously unable to tolerate ND0612 and/or had experienced intolerable adverse drug reactions associated with its use, regardless of the dosing regimen administered.
Male and female subjects with PD, of any race, aged ≥30 years who have provided written informed consent.	Atypical or secondary parkinsonism.
PD diagnosis consistent with the UK Brain Bank Criteria.	Acute psychosis or hallucinations in the 6 months preceding enrollment.
Modified Hoehn & Yahr scale ≤3 during ON.	Prior neurosurgical procedure for PD, or Duopa/Duodopa treatment.
Taking ≥4 doses/day of LD/DDI (or ≥ 3 doses/day of Rytary) and taking, or have attempted to take, ≥1 other PD treatment for at least 30 days.	Any malignancy in the 5 years prior to randomization (excluding basal cell carcinoma of the skin or cervical carcinoma in situ that had been successfully treated).
Patients had to be stable on their anti-PD medications for ≥30 days before Day 1.	Positive serum serology for Hepatitis B Virus (HBV), Hepatitis C Virus (HCV) or Human Immunodeficiency Virus (HIV) at the Screening visit.
Patients could have had prior exposure to SC apomorphine injections/infusion but had to have stopped continuous apomorphine administration ≥4 weeks before the screening visit. Treatment with apomorphine was prohibited during the entire ND0612 treatment period.	Any relevant medical, surgical, or psychiatric condition, laboratory value, or concomitant medication which, in the opinion of the Investigator made the subject unsuitable for study entry or potentially unable to complete all aspects of the study.
Minimum of 2 hours of OFF time per day with predictable early morning OFF periods as estimated by the patient.	Patients with a history of drug abuse or alcoholism within the past 12 months.
Predictable and well-defined early morning OFF periods with a good response to LD for treatment of the early morning OFF in the judgment of the Investigator.	Clinically significant ECG rhythm abnormalities.
Mini-Mental State Examination (MMSE) score ≥26.	Renal or liver dysfunction that could alter drug metabolism including: serum creatinine >1.3 mg/dL, serum aspartate aminotransferase (AST) or alanine aminotransferase (ALT) >2 x upper limit of normal (ULN), total serum bilirubin >2.5 mg/dL.
No clinically significant medical, psychiatric or laboratory abnormalities which the Investigator judged would be unsafe or non-compliant in the study.	Current participation in a clinical trial with an investigational product or past participation within the last 30 days before Day 1.
Female patients had to be surgically sterile (hysterectomy, bilateral oophorectomy, or tubal ligation), postmenopausal (defined as cessation of menses for at least 1 year), or willing to practice a highly effective method of contraception.	
Willing and able to administer the SC infusion alone or with the assistance of a study partner after a screening period of up to 40 days and willing and able to comply with study requirements.	
Named study partner.	

Supplementary Figure e1. Disposition of Subjects by Dosing Regimen at 1 year

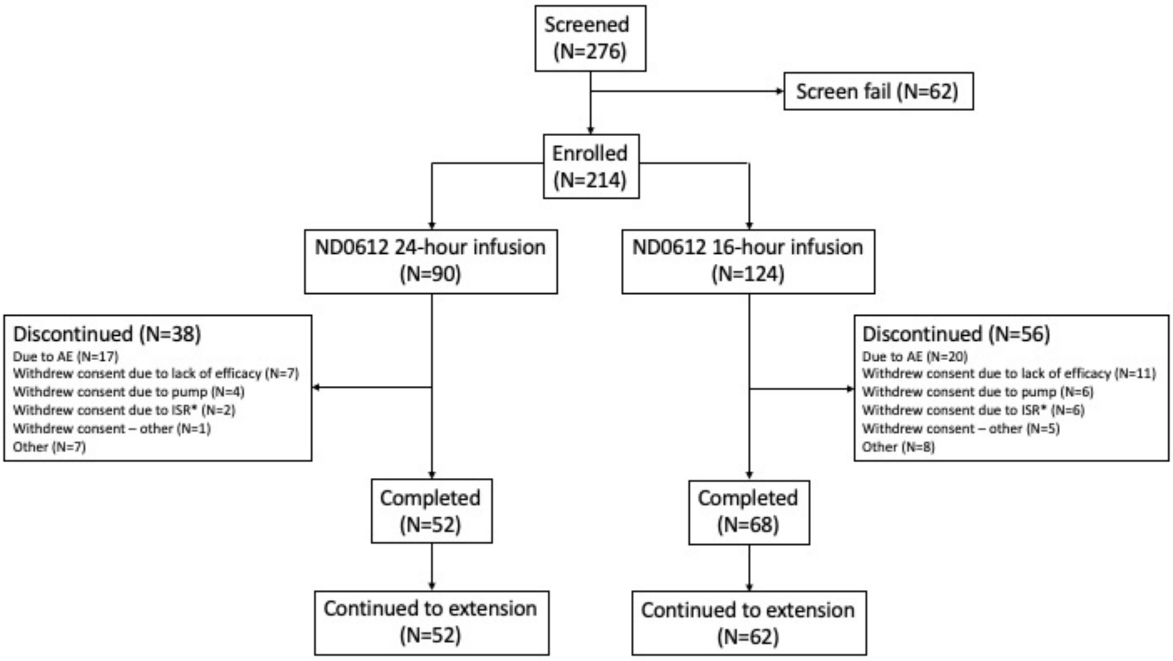

Discontinuation rates improved during study conduct following a protocol amendment, from 49% to 29%.

Supplementary Figure e2. Examples of nodules and hematomas after 12 months infusion with ND0612. (A) Patient with a few nodules and hematomas (B) Patient with more nodules and hematomas.

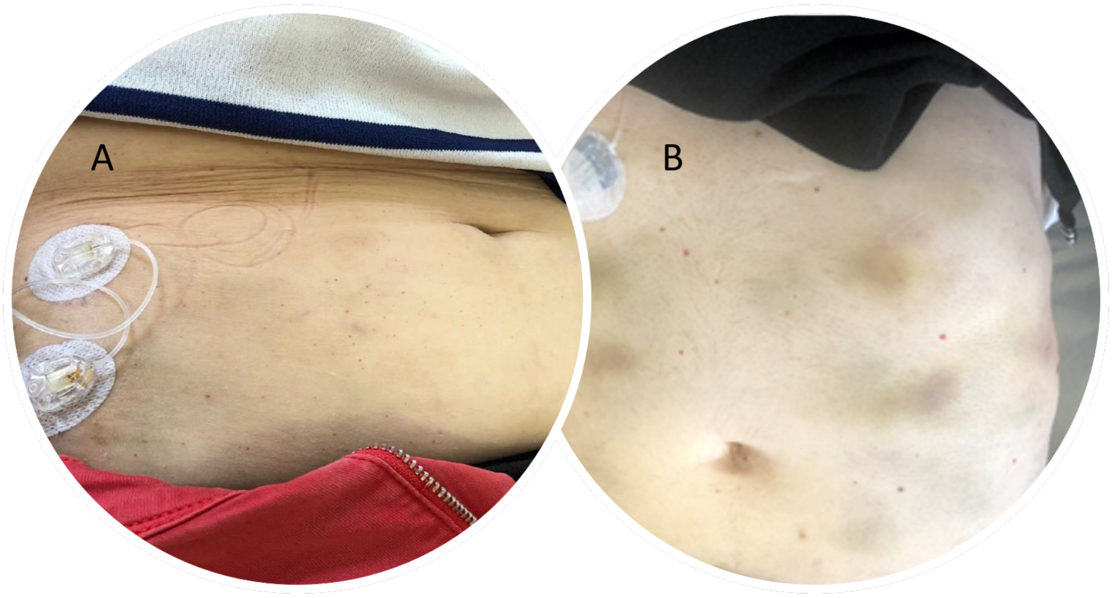

Although infusion site reactions (ISRs) were common, they were generally mild and led to discontinuation in only 10%. ISRs are an expected feature of continuous drug delivery via the subcutaneous route, and especially in PD where skin disorders are already common. Experience with subcutaneous apomorphine delivery shows that it is vital to set expectations of treatment for the patient, caregiver, and physicians, such that they are not fazed by the appearance of nodules and other ISR and have an understanding of their practical management. In this study, while the majority of patients developed skin nodules, these were only reported as an AE in a third of cases when considered clinically significant by the investigator.

Supplementary Figure e3. Exploratory efficacy measures (a) Good ON time and (b) OFF time (c) UPDRS scores and (d) Clinical Global Impression scores

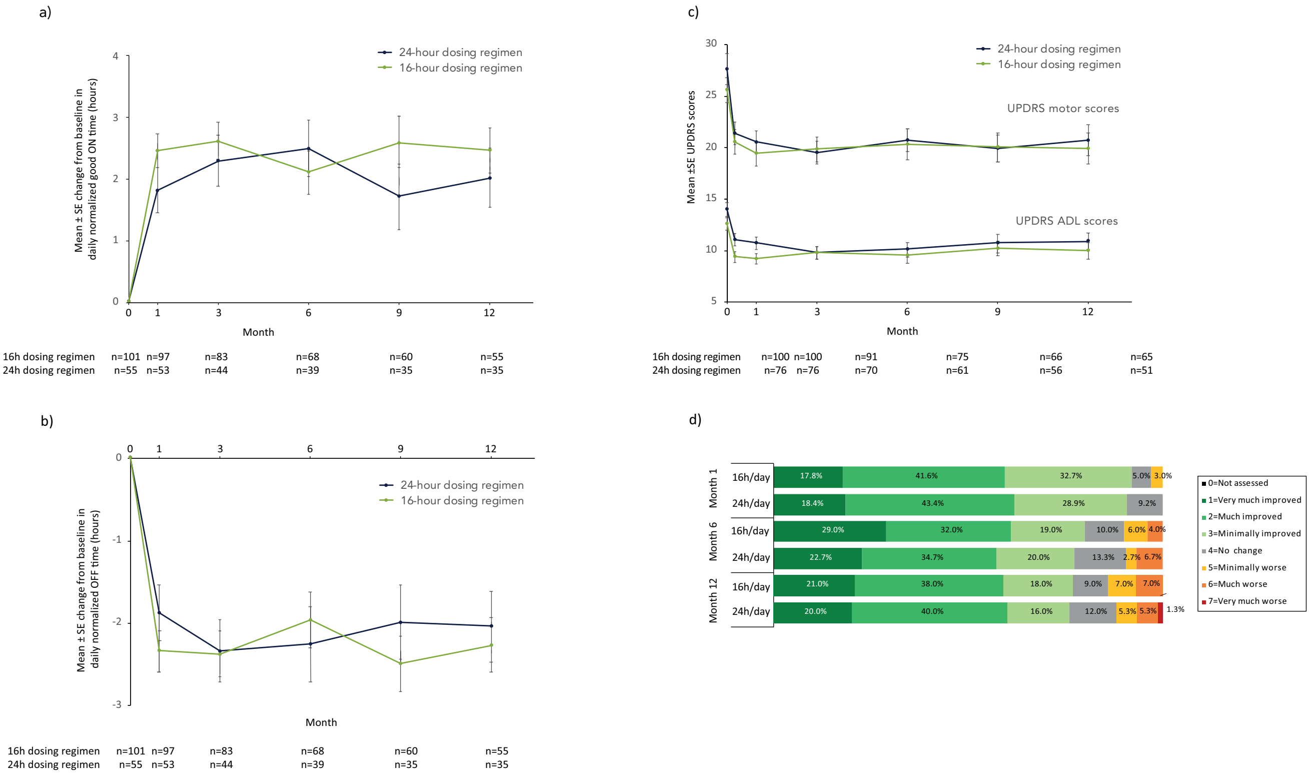

Exploratory efficacy measures were evaluated using a Mixed Model for Repeated Measures (MMRM), including treatment regimen, visit, and the interaction between treatment regimen and visit as fixed factors and the Baseline value as covariate. Changes in CGI scores were assessed using the last observation carried forward (LOCF).
